# COVID-19 Prediction With Machine Learning Technique From Extracted Features of Photoplethysmogram Morphology

**DOI:** 10.3389/fpubh.2022.920849

**Published:** 2022-07-19

**Authors:** Nazrul Anuar Nayan, Choon Jie Yi, Mohd Zubir Suboh, Nur-Fadhilah Mazlan, Petrick Periyasamy, Muhammad Yusuf Zawir Abdul Rahim, Shamsul Azhar Shah

**Affiliations:** ^1^Faculty of Engineering and Built Environment, Universiti Kebangsaan Malaysia, Bangi, Malaysia; ^2^Institute Islam Hadhari, Universiti Kebangsaan Malaysia, Bangi, Malaysia; ^3^Institute for Environment and Development, Universiti Kebangsaan Malaysia, Bangi, Malaysia; ^4^UKM Medical Centre, Hospital Canselor Tuanku Muhriz, Cheras, Malaysia; ^5^Faculty of Medicine, UKM Medical Centre, Cheras, Malaysia

**Keywords:** COVID-19, photoplethysmogram, machine learning, non-invasive, diagnostic, prediction

## Abstract

At present, COVID-19 is spreading widely around the world. It causes many health problems, namely, respiratory failure and acute respiratory distress syndrome. Wearable devices have gained popularity by allowing remote COVID-19 detection, contact tracing, and monitoring. In this study, the correlation of photoplethysmogram (PPG) morphology between patients with COVID-19 infection and healthy subjects was investigated. Then, machine learning was used to classify the extracted features between 43 cases and 43 control subjects. The PPG data were collected from 86 subjects based on inclusion and exclusion criteria. The systolic-onset amplitude was 3.72% higher for the case group. However, the time interval of systolic-systolic was 7.69% shorter in the case than in control subjects. In addition, 12 out of 20 features exhibited a significant difference. The top three features included dicrotic-systolic time interval, onset-dicrotic amplitude, and systolic-onset time interval. Nine features extracted by heatmap based on the correlation matrix were fed to discriminant analysis, k-nearest neighbor, decision tree, support vector machine, and artificial neural network (ANN). The ANN showed the best performance with 95.45% accuracy, 100% sensitivity, and 90.91% specificity by using six input features. In this study, a COVID-19 prediction model was developed using multiple PPG features extracted using a low-cost pulse oximeter.

## Introduction

The most recent threat to global health is the ongoing outbreak of the respiratory disease since 2019, namely, Coronavirus disease 2019 (COVID-19), which originates from the initial cases reported in Wuhan, China (1–3). This novel disease is the seventh member of the family of coronaviruses that can infect humans (4, 5). Patients with COVID-19-infection typically exhibit symptoms such as fever, dry cough, and shortness of breath. Severe COVID-19 infection could progress to pneumonia, respiratory failure, and acute respiratory distress syndrome (6, 7). The highly contagious nature and unavailability of a specific cure for COVID-19 infection have led to various detection methods over the past 3 years.

Apart from reverse transcription-PCR (RT-PCR) confirmation, researchers have utilized X-ray and CT imaging methods for COVID-19 screening or diagnostic purposes. To date, many researchers have developed automated X-ray imaging to detect pneumonia, distinguish it from non-COVID-19 pneumonia, and predict its severity and progression (8–12). Automated CT imaging has been studied to detect not only lesions but also pneumonia. It recognizes pneumonia symptoms and distinguishes it from influenza-A viral pneumonia and healthy patient (13–16).

The reading of vital signs, such as oxygen saturation (SpO_2_), heart rate (HR), blood pressure, body temperature, respiration rate (RR), and glucose concentration, as an initial diagnostic measure is vital in identifying medical issues (17–19). A Wi-COVID, a home-based Wi-Fi monitoring, has been developed to monitor the RR of patients at home. Although it allows real-time monitoring, the application was limited to home environment setting only (20). In another research, a deep learning-Raspberry Pi integration was based on cough detection of a patient with COVID-19. However, this study only displayed the contact tracing solution as an extension to conventional presence detection and person identification systems. It does not help recognize the COVID-19 cough immediately from a potentially infected person (21). A contact tracing app with Bluetooth low-energy compatible devices has been evaluated for COVID-19 proximity detection by considering the signal strength caused by the human body and other factors. Nonetheless, received signal strength measurements are easily influenced, i.e., they fluctuate substantially depending on the absorption by the human body, relative handset orientation, and radio signal absorption or reflection in buildings and trains (22).

Wearable devices have gained popularity as they allow remote COVID-19 detection, contact tracing, and monitoring, for example, home, medical ambulance, ambulatory, emergency, workplace, and hospital (18). An IoT-based sensor to identify and monitor isolated asymptotic patients has been reported. However, the proposed system lacks clinical data for comparison using actual COVID-19 samples (23). A headset has been used in combination with a mask, such as a thermistor being embedded in the earphone, whereas an HR sensor equipped with the ear clip has been devised. Even though the principle was based on a simple and low-cost component, the mask is no longer mandatory in public following the implementation of the vaccination program, and long-term use could cause skin problems such as mask acne or maskne (24, 25). The smart helmet based on the thermal imaging system for real-time monitoring of body temperature and global positioning system *via* face recognition and mobile phones has been developed. As fever is one of the common COVID-19 symptoms, other viral infections can also exhibit high body temperature and are hardly distinguishable (26, 27).

Photoplethysmogram (PPG), has been utilized widely because of its non-invasiveness, simplicity, and affordability (28, 29). This technique is advantageous in measuring blood volume changes per pulse optically at the skin surface (30). The optical measurement comprises red and infrared lights as a light source and a photodetector, which mechanically indicates the heart activity (31, 32). Therefore, as a non-invasive and convenient technique, PPG can play a significant role in health monitoring systems to continuously provide various health parameters such as HR, RR, and SpO_2_ (33). In the case of COVID-19, the use of PPG is paramount in reducing widespread infection during health monitoring of patients by promoting lesser skin contact and abolishing the need to collect their bodily fluids invasively from time to time for checking progress. Currently, fingertip pulse oximeters are widely used by medical practitioners to measure SpO_2_ in patients with COVID-19 owing to their portability and inexpensiveness (34). Moreover, pulse oximeters are easily accessible with no hassle in designing a new device or further addition of an extra layer to enable detection.

People doing COVID-19 screening at hospitals are also at risk of being exposed to the virus, especially when there is a new strain outbreak which usually causes overcrowding of hospitals. Nevertheless, the commercially available self-diagnostic kits require specialized collection tubes that are non-reusable and expensive, which can be burdensome in the long run. Therefore, there is a need for accurate, rapid, portable, reusable, and easy-to-administer diagnostic tools to help communities manage local outbreaks and assess the spread of disease. In this study, the correlation of PPG morphology between patients with COVID-19-positive and negative was investigated. The PPG signals were pre-processed using a Fourier transform technique and significantly extracted features were fed to machine learning algorithms for COVID-19 classification purposes.

## Methodology

### PPG Data Acquisition

For the case group, the inclusion criteria were as follows: RT-PCR-positive subjects, categories 3 and 4 of infection, admitted to a ward, and aged between 18 and 65 years old. Meanwhile, the inclusion criteria for the control group were as follows: RT-PCR-negative subjects with ages ranging from 18 to 65 years old. For both groups, the exclusion criteria were as follows: pregnant, smoking, and having cardiovascular-related diseases.

This study used a case-control design whereby only a one-time measurement for each sample was performed. The subjects were recruited until the number shown in the sample size calculation was achieved. A simple way to determine the sample size is by referring to a nomogram where standardized difference and power calculation indicate the sample size (35). The system aimed to achieve at least 95% sensitivity (SN) with a CI (W) of 0.15. The COVID-19 prevalence among the Malaysian population by January 31st, 2022, is 9.4% (P), which was based on the calculation of 3,042,780 infected patients. As shown in **Equations (1), (2)**, the estimated total number of samples was 86; thus, 43 subjects were involved in each case and control group, respectively.


(1) $$\begin{array}{l} TP+FN=\frac{Z^{2}\left(SN\left(1-SN\right)\right)}{W^{2}}\\ \;=\;\frac{1.96^{2}\left(0.95\left(1-0.95\right)\right)}{0.15^{2}}=8.11 \end{array}$$

(2) $$\begin{array}{l} Sample\;Size,\;N\left(Sensitivity\right)=\;\frac{TP+FN}{P}\\ \;=\;\frac{8.11}{0.094}\approx 86\;Samples \end{array}$$

Where:

*TP* = true positive
*FN* = false negative
*Z* = standard score
*W* = confidence interval
*P* = prevalence

**Figure 1:**
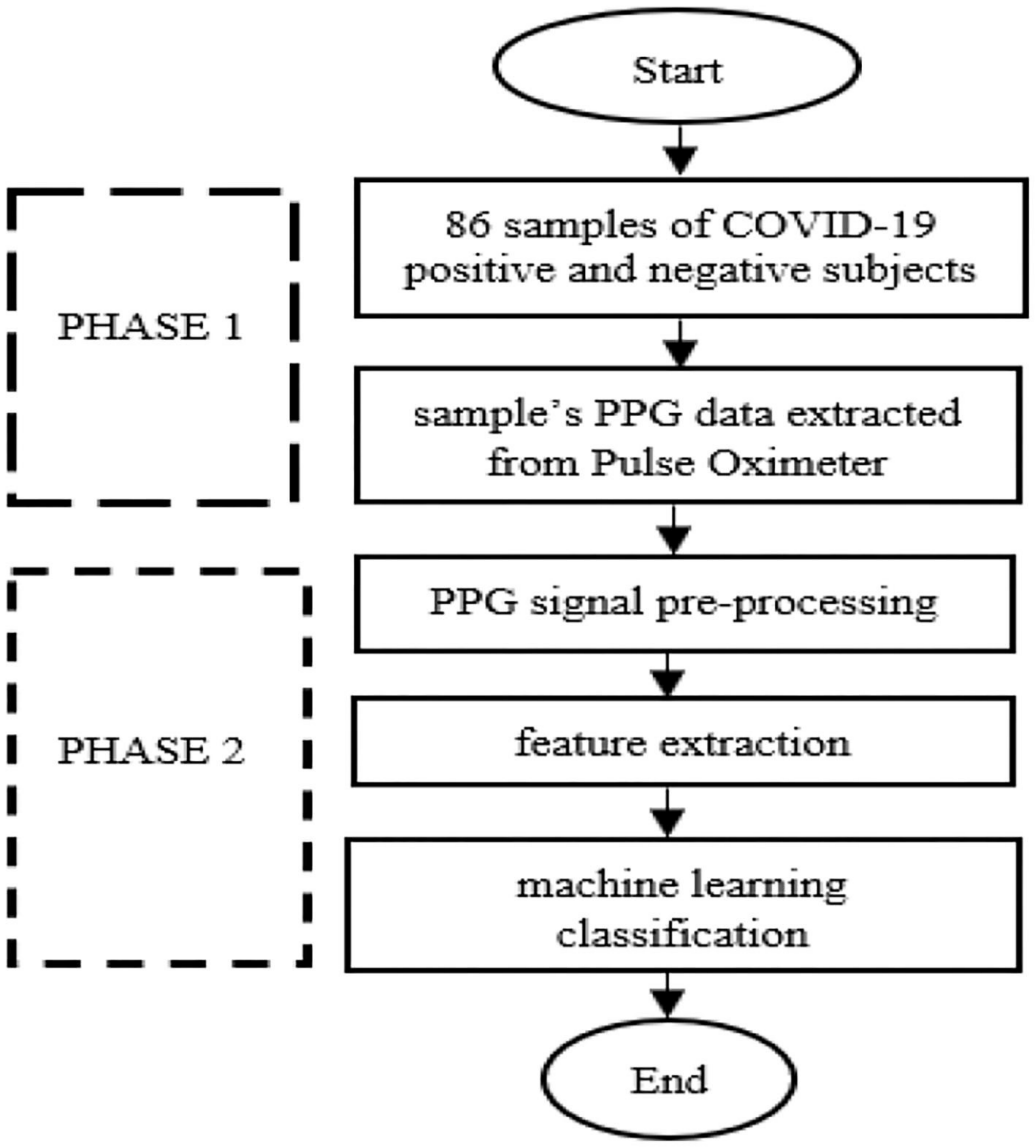
Methodology in developing COVID-19 prediction model by utilizing photoplethysmogram.

**Figure 2:**
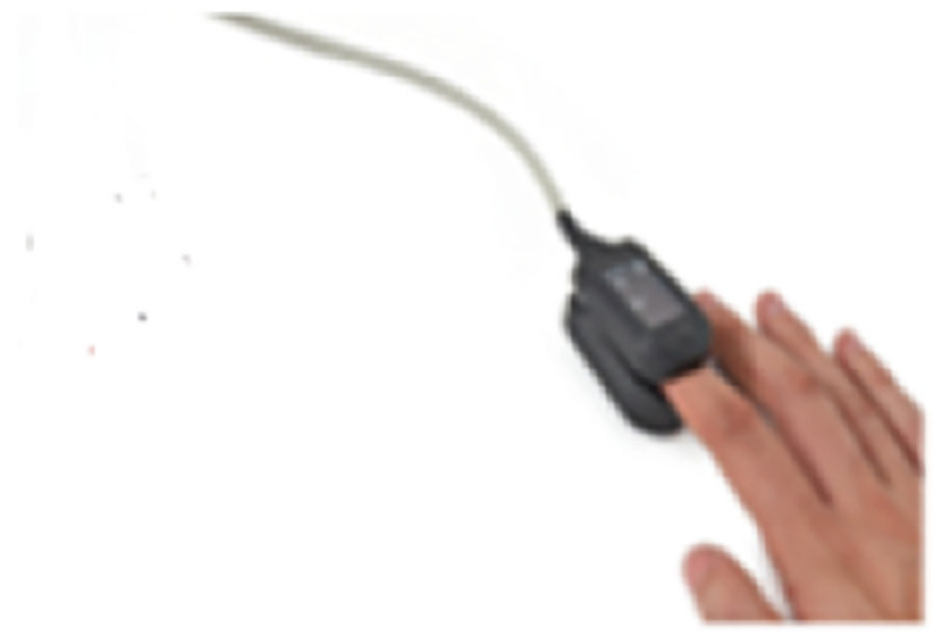
PPG data taken from the index finger of the right hand during a 10-min resting period.

Figure 1 shows the methodology for developing a COVID-19 prediction model by utilizing PPG. Before PPG signal measurement, 43 case subjects were in a supine position where they lay down horizontally with the face and torso facing up in a relaxed condition. By contrast, the 43 control subjects were in a sitting position and had quiet manners. All data were collected from the index finger of the right hand during a 10-min resting period (Figure 2). Data collection was performed using a pulse oximeter (CMS50D+, Contec, China) at a sampling frequency of 100 Hz. A trained medical officer recorded data collection from case samples by pushing the start button on the pulse oximeter and laptop after placing the pulse oximeter on the right index finger. The same method was also used for 43 healthy samples of the control group, where data collection was performed outside of Hospital Canselor Tuanku Muhriz, Universiti Kebangsaan Malaysia (HCTM, UKM). All researchers involved wore a PPE set during data collection. A thorough sanitization using a pulse oximeter and laptop was performed after each collection.

### Pre-processing and Signal Quality Indexing

Photoplethysmogram signal pre-processing algorithm consists of baseline and high-frequency removal and signal quality indexing (SQI). All PPG data were processed offline in MATLAB. In this step, a fast Fourier transform technique was used as a band-pass filter with a cut-off frequency of 0.5–10.0 Hz. Based on the collected PPG signals, a frequency above 10 Hz was considered high-frequency noise, and that below 0.5 Hz was attributed to baseline wander. Amplitude offset was found using filtering. Auto-offsetting was used to bring back any y-axis below zero value to a positive value to address this problem. It was performed by offsetting the signal by the difference between zero-amplitude and the prominent negative value.

**Figure 3:**
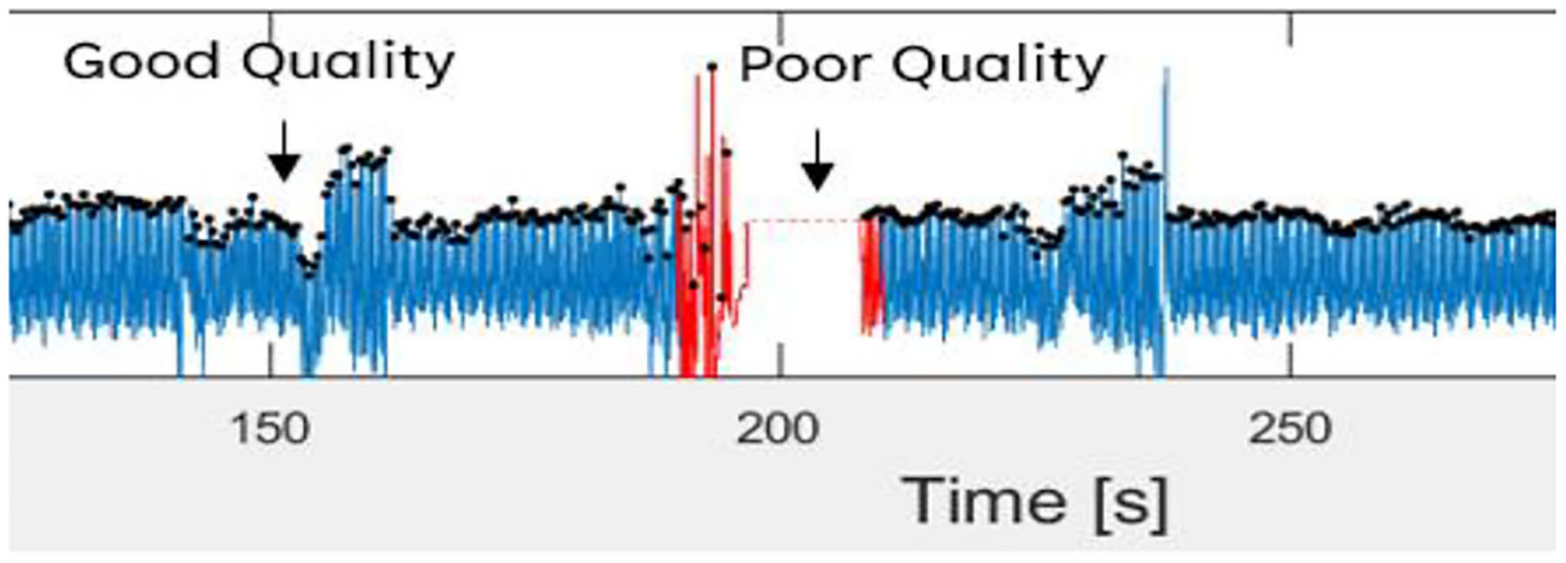
Good and poor quality of PPG signal as defined in blue and red color signal, respectively.

Then, the filtered PPG signal underwent SQI in determining reliable signals. This step is crucial because only a high-quality signal is processed for the next phase. This type of signal is referred to as a stable signal within a period of time, in which the three conditions proposed by (36) are achieved as follows: (1) the extrapolated 10-s PPG signal must be between 40 and 180 bpm; (2) the PPG pulse-peak gap must not exceed 3 s to avoid missing more than one beat; and (3) the ratio of the maximum and minimum beat-beat interval within a sample must be less than 2.2. Figure 3 shows poor and good-quality PPG signals after SQI, as illustrated in red and blue colors, respectively.

### Feature Extraction

**Figure 4:**
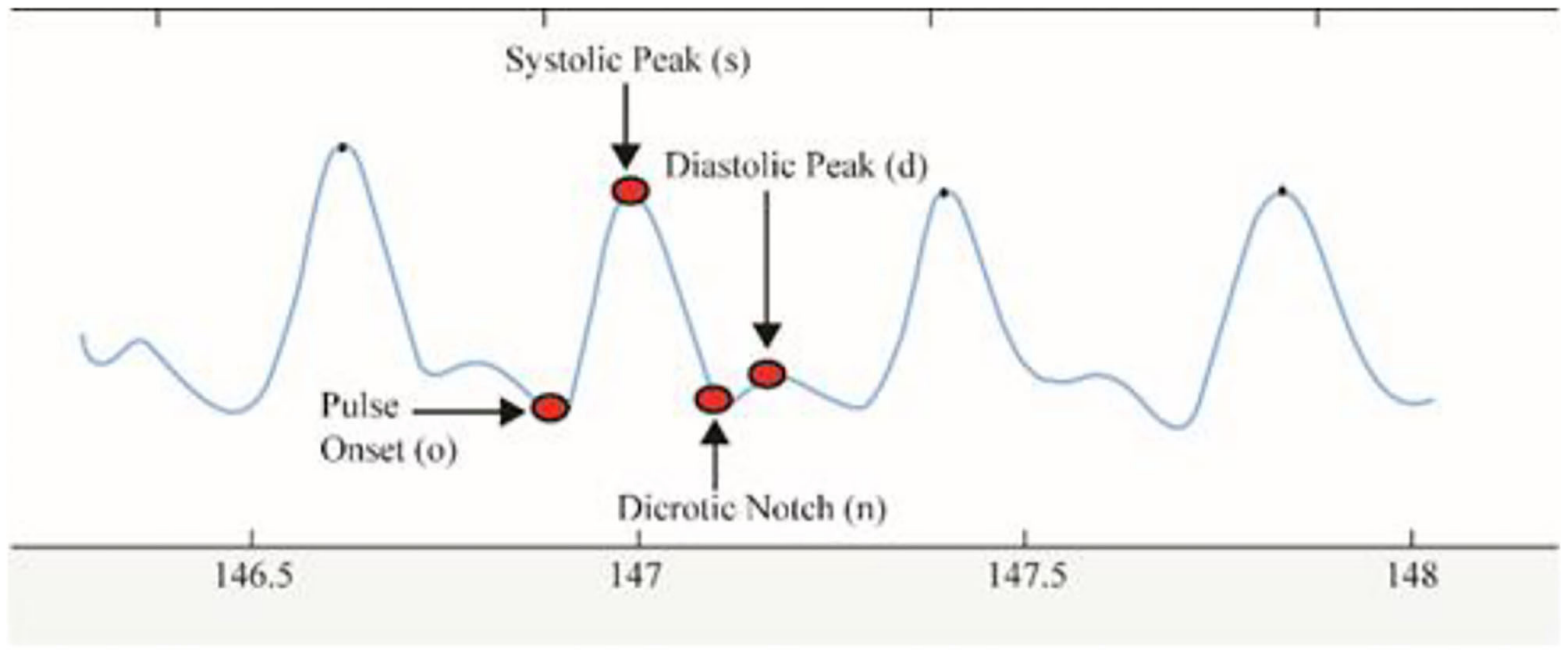
PPG signal showing all the fiducial points.

*Delineator* (37) and *bp_annotate* (38) algorithms were applied to detect the fiducial points in the PPG signals. These points include pulse onset “o,” systolic peak “s,” dicrotic notch “n,” and diastolic peak “d” (Figure 4). Both algorithms were compared manually in detecting fiducial points toward our PPG data. The *delineator* algorithm accurately detects the o and s, and *bp annotate* is good at detecting the peaks of n and d. The determination of pulse onset related to the zero-crossing point before maximum inflection and the s peak was defined as the zero-crossing point after inflection (37). Table 1 shows the features used in the proposed method. o2s represents onset-systolic, and this nomenclature applies to all other fiducial points. hr expresses the amplitude, and wt refers to the time interval measured in seconds.

**Table 1:** PPG fiducial points and the features.

**PPG fiducial point**	**Feature label**
Onset	o2o_wt, o2s_wt, o2n_wt, o2d_wt, o2s_hr, o2n_hr, o2d_hr, s2n_hr
Systolic	s2s_wt, s2o_wt, s2n_wt, s2d_wt
Notch	n2n_wt, n2s_wt, n2o_wt, n2d_wt
Diastolic	d2d_wt, d2o_wt, d2s_wt, d2n_wt

### Statistical Analysis

A total of 20 features were obtained from PPG. Descriptive statistics in ranking the features based on the *P* value was applied by analyzing the mean values from both case and control data. The normality of the distribution was assessed using the Kurtosis and Skewness tests by combining case and control groups for each feature. The value of asymmetry, that is, Skewness and Kurtosis between −2 and +2, was considered acceptable to prove normal univariate distribution (39). Non-normally distributed features underwent Mann–Whitney *U* or Wilcoxon rank-sum test by using MATLAB to find the significant difference between patients with COVID-19-positive and negative, by comparing the median between the two groups. An independent *t*-test was also performed for normally distributed features using MATLAB where means were compared between the two groups. The features were then sorted by the lowest to the highest *P* values with a CI of 95%. This step enhances the accuracy rate of the classifiers (40). Significant features (*P* < 0.05) were used as inputs for ML classification.

Features with a *P* value <0.05 were evaluated using a correlation matrix for feature selection. Similarities among features of each group were determined using the Pearson correlation co-efficient, and *r* was calculated using **Equation (3)**. The *r* value shows the correlation Co-efficient between two variables, where a value near 0 indicates no correlation, whereas a value near 1 or −1 indicates a strong correlation, positively or negatively, between the two variables. If two features have high correlation strength with absolute correlation Co-efficient, then |*r*| > 0.8; both features share similar information. This result could increase the complexity of ML training and reduce its performance. Thus, only the features with high correlation strength were selected for analysis.


(3) $$r\;=\;\frac{\sum_{i=1}^{N}\left(x_{i}-\hat{x})(y_{i}-\hat{y}\right)}{\sqrt{\sum_{i=1}^{N}{\left(x_{i}-\hat{x}\right)}^{2}\sum_{i=1}^{N}{\left(y_{i}-\hat{y}\right)}^{2}}}$$

Where:

*x* = first feature variables
*y* = second feature variables or group variables
$\hat{x}$ = mean of feature *x*
ŷ = mean of feature *y* or mean of group *y*
*N* = number of samples

### Machine Learning

The final phase of the proposed method aimed to design an effective decision boundary to classify two groups of case and control. Supervised ML techniques such as discriminant analysis (DA), k-nearest neighbor (KNN), decision tree (DT), support vector machine (SVM), and artificial neural network (ANN) were used in this study because they are the commonly used classifiers for biomedical signal analysis (41, 42).

Discriminant analysis (DA) is commonly selected as the first classifier because it is fast and easy to interpret. Linear and quadratic types of DA were used in this study. KNN is another simple and early classification algorithm (43). The algorithm counts the nearest neighbors (*k*), and classification is performed in a “vote” form. The *k* value was set from 1 to 100 because different *k* could produce different classification results. Euclidean distance measurement was used. Meanwhile, DT is a notable algorithm where decision logics are performed based on the outcomes in a tree-like structure, from the beginning nodes (root) down to the leaf node. Gini's diversity index was used in this study, and the split number was set from 1 to 100.

Apart from DA, KNN, and DT, SVM can supervise classification problems because of its generalization capability (44). SVM optimizes the decision boundary, that is, hyperplane, to obtain a significant separating margin in a higher feature dimension. In this study, SVM with four different kernel functions was tested with fixed hyperplane parameters. The selected kernels include linear and radial basis functions, and third and fourth-order polynomial kernels. On the contrary, ANN is well known for pattern recognition and classification. Three layers of a feed-forward multilayer perceptron network were constructed with different hidden nodes at each hidden layer. The Levenberg–Marquardt training algorithm was applied during training using “*log-sigmoid*” and “*purelin*” activation functions.

These ML models were trained with 64 train data of around 75% of total data, and the other 22 were used as test data. The data were randomly divided and stratified into groups. Features obtained from correlation analysis with *P* < 0.05 were selected as the input for the ML model. Features were cumulatively fed one by one starting with the least *P* value. The target was set in binary of “0” and “1” for the control and case groups, respectively. The training was validated using fivefold cross-validation, where a subfold of 13 from the 64-train data was used to validate the model. The performance of each model was measured based on mean squared error (MSE), specificity (SP), SN, and accuracy (ACC). A comparison of the suitable ML model on each technique was performed using receiver operating characteristic curves (ROC), where the false positive rate (FPR) and true positive rate (TPR) of the trained model were recorded with a discriminant threshold of 0–1. The area under the curve of the ROC was compared, where an area under the curve (AUC) value closer to 1 indicated a better predictor model.

**Figure 5:**
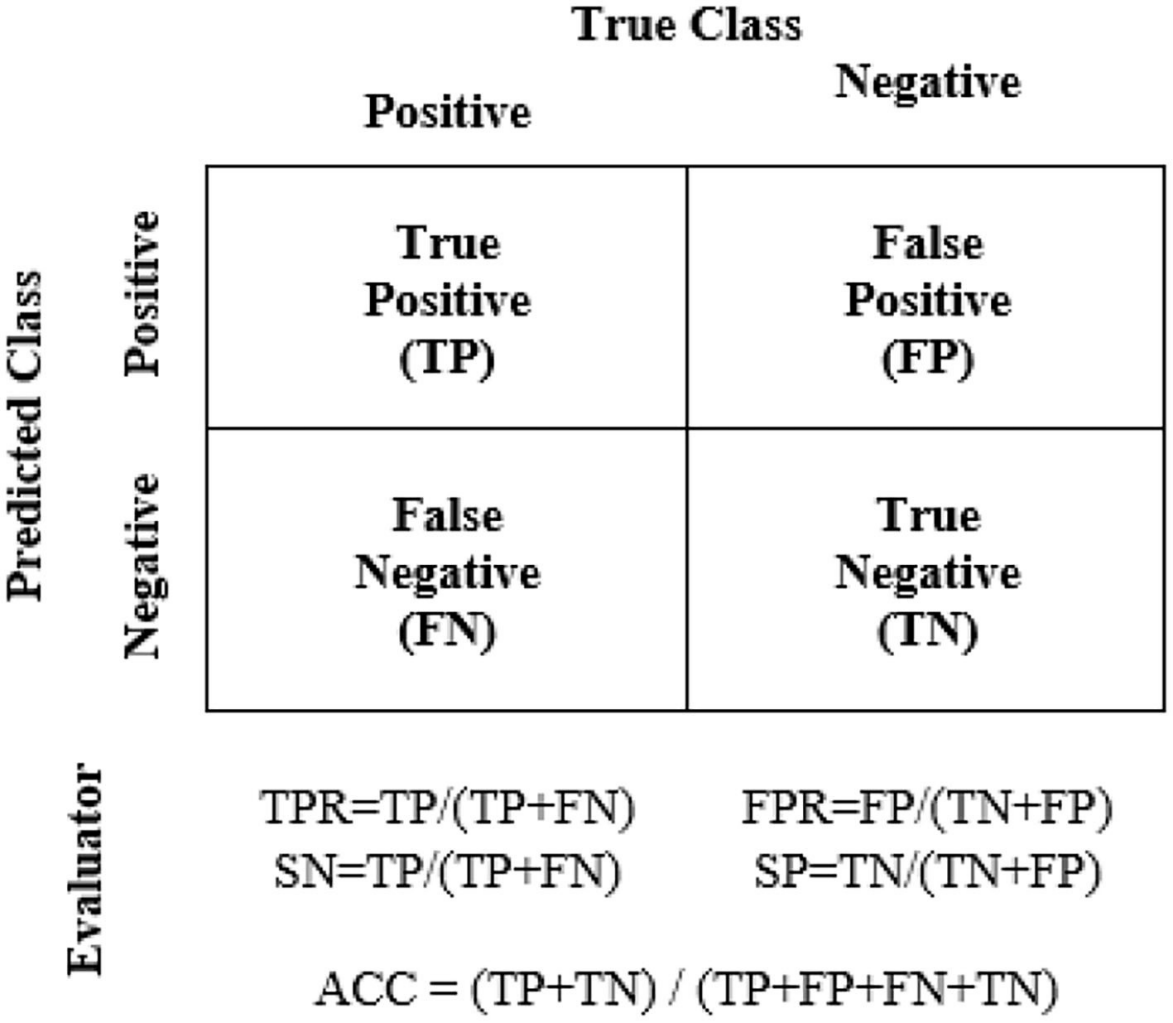
Performance evaluators for ML-trained models.

The performance evaluators are shown in Figure 5 where true positive (TP) = number of positive COVID-19 was accurately detected as the case group, true negative (TN) = number of negative COVID-19 as the control group, false negative (FN) = number of positive COVID-19 as the control group, and false positive (FP) = number of negative COVID-19 as the case group. This study was conducted in accordance with the Declaration of Helsinki, in which the protocol was approved by the Research and Ethics Committee of the HCTM with a registration number of UKM PPI/111/8/JEP-2020-828.

## Result and Discussion

### PPG Data Acquisition

A total of 43 case subjects from HCTM and 43 control subjects from the public volunteered for data collection. The demographic information of 86 subjects was categorized (Table 2). The study population involved 86 subjects (46 men and 40 women), with two different groups of subjects: case and control. All volunteers submitted their informed consent before data collection. A questionnaire form consisting of the health status of the subjects was provided.

**Table 2:** Descriptive characteristics of the subjects (*N* = 86).

**Characteristics**	**Mean (SD)**	
	**Case** **(*N* = 43)**	**Control** **(*N*= 43)**
Age (years), Mean(SD)	57.93 (13.75)	58.65 (13.82)
Gender	23 M/20 F	23 M/20 F
Ethnic	21 Malays	24 Malays
	18 Chinese	18 Chinese
	2 Indians	0 Indians
	1 Pakistani	0 Pakistani
	1 Indonesian	1 Indonesian

In the case group, two stages of patients with COVID-19 were involved, stages 3 and 4. A total of 72% of subjects had comorbidities, such as diabetes mellitus, hypertension, and chronic kidney disease. Most of the case subjects with COVID-19 had comorbidities. The data of the case group were recorded from 23 male and 20 female patients of different ethnic groups consisting of 21 Malays, 18 Chinese, two Indians, one Pakistani, and one Indonesian with ages ranging from 29 to 89 years old with a mean age of 57.93 (SD = 13.75).

Meanwhile, the control group data were recorded from 23 men and 20 women of different ethnic groups, namely, 24 Malays, 18 Chinese, and one Indonesian, aged 29–87 years old with a mean age of 58.65 (SD = 13.82). Filtering from 86 sets of high-quality PPG signals resulted in 20 extracted features with 10-min recording per subject. These extracted features were based on four fiducial points mentioned in the Methodology section.

### Pre-processing and Signal Quality Indexing

**Figure 6:**
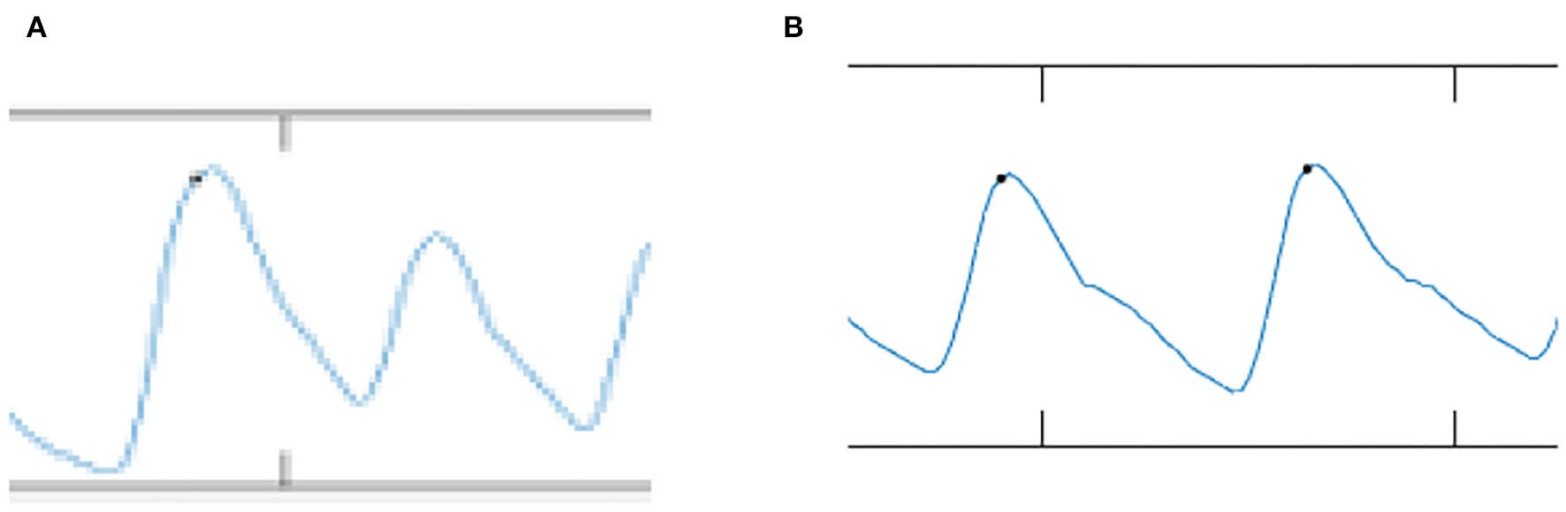
Comparison of PPG signal between **(A)** case and **(B)** control group.

Figure 6 shows the comparison of the PPG signal between case and control subjects. For the PPG signal of case subjects as shown in Figure 6A, the inflection points of o, s, n, and d peaks are smaller. By contrast, for control subjects (Figure 6B), the inflection points of o, s, n, and d signals were normal, clear, and significant. The s2o_hr is 3.72% higher in case subjects than in control subjects. The time interval of s2s_wt was 7.69% shorter in case subjects than in control subjects.

### Feature Extraction

These 20 features were extracted by using MATLAB and recorded in Table 3, where significant *P*-values are written in bold. As shown in Table 3, 12 out of 20 features were identified with a significant *P* value < 0.05 and were fed as inputs for the ML algorithm. The features with the highest *P*-value included d2s_wt, o2n_hr, and s2o_wt. The features were fed to machine learning models, namely, DA, KNN, DT, SVM, and ANN.

**Table 3:** Feature extraction of case and control ranked based on the *P* value.

**No**	**Feature (median)**	**Mean (SD)**		***P*-value**
		**Case** **(*N* = 43)**	**Control** **(*N* = 43)**	
1	**d2s_wt**	**0.34 (0.06)**	**0.38 (0.07)**	**1.0238** **×10**^**−17**^
2	**o2n_hr**	**17.60 (6.65)**	**21.49 (5.32)**	**2.7352** **×10**^**−17**^
3	**s2o_wt**	**0.32 (0.08)**	**0.37 (0.07)**	**9.5081** **×10**^**−17**^
4	**n2s_wt**	**0.32 (0.08)**	**0.36 (0.08)**	**6.2534** **×10**^**−15**^
5	**o2d_hr**	**18.84 (7.88)**	**23.48 (5.00)**	**4.1973** **×10**^**−12**^
6	**s2s_wt**	**0.48 (0.08)**	**0.52 (0.08)**	**5.3208** **×10**^**−12**^
7	**d2d_wt**	**0.48 (0.09)**	**0.52 (0.08)**	**2.1099** **×10**^**−11**^
8	**s2n_hr**	**20.83 (9.24)**	**17.73 (6.69)**	**1.3851** **×10**^**−8**^
9	**s2d_wt**	**0.14 (0.04)**	**0.16 (0.05)**	**7.5295** **×10**^**−7**^
10	**n2d_wt**	**0.21 (0.16)**	**0.23 (0.19)**	**1.8000** **×10**^**−3**^
11	**d2o_wt**	**0.20 (0.06)**	**0.22 (0.05)**	**2.3380** **×10**^**−4**^
12	**o2s_wt**	**0.15 (0.05)**	**0.15 (0.03)**	**0.01717**
13	n2n_wt	0.59 (0.44)	0.54 (0.12)	0.05662
14	o2o_wt	0.59 (0.43)	0.54 (0.13)	0.6368
15	o2s_hr	33.14 (10.36)	31.95 (6.58)	0.2209
16	o2n_wt	0.30 (0.09)	0.31 (0.07)	0.2479
17	o2d_wt	0.28 (0.04)	0.31 (0.09)	0.2657
18	n2o_wt	0.33 (0.51)	0.22 (0.11)	0.2931
19	d2n_wt	0.26 (0.19)	0.25 (0.20)	0.3687
20	s2n_wt	0.15 (0.07)	0.37 (0.07)	0.6301

*The 12 bold items indicates 12 statistically significance features with P-value of less than 0.05 (p < 0.05)*.

### Statistical Analysis

Normality tests such as Kurtosis and Skewness are shown in Table 4 with normally distributed features written in bold.

**Table 4:** Normality tests of kurtosis and skewness.

**No**	**Feature (median)**	**Kurtosis value**	**Skewness value**
1	**s2s_wt**	**-1.0355**	**0.3945**
2	**s2o_wt**	**-0.3979**	**0.5488**
3	s2n_wt	12.9306	2.6589
4	**s2d_wt**	**1.4588**	**0.8479**
5	o2o_wt	35.5860	5.4855
6	o2s_wt	32.5283	4.8299
7	o2n_wt	3.3604	**1.5140**
8	o2d_wt	31.3579	4.3775
9	n2n_wt	31.0130	5.5323
10	**n2s_wt**	**0.05718**	**-0.0429**
11	n2o_wt	32.7718	5.5978
12	**n2d_wt**	**-1.4380**	**0.3839**
13	**d2d_wt**	**-0.9702**	**0.4085**
14	**d2o_wt**	**-0.4939**	**0.7442**
15	**d2s_wt**	**-0.8768**	**0.5192**
16	**d2n_wt**	**-1.5927**	**0.2196**
17	o2s_hr	2.2197	**-0.9983**
18	**o2n_hr**	**0.4237**	**-0.3175**
19	**o2d_hr**	**0.1843**	**-0.5749**
20	**s2n_hr**	**0.4694**	**0.8305**

**Figure 7:**
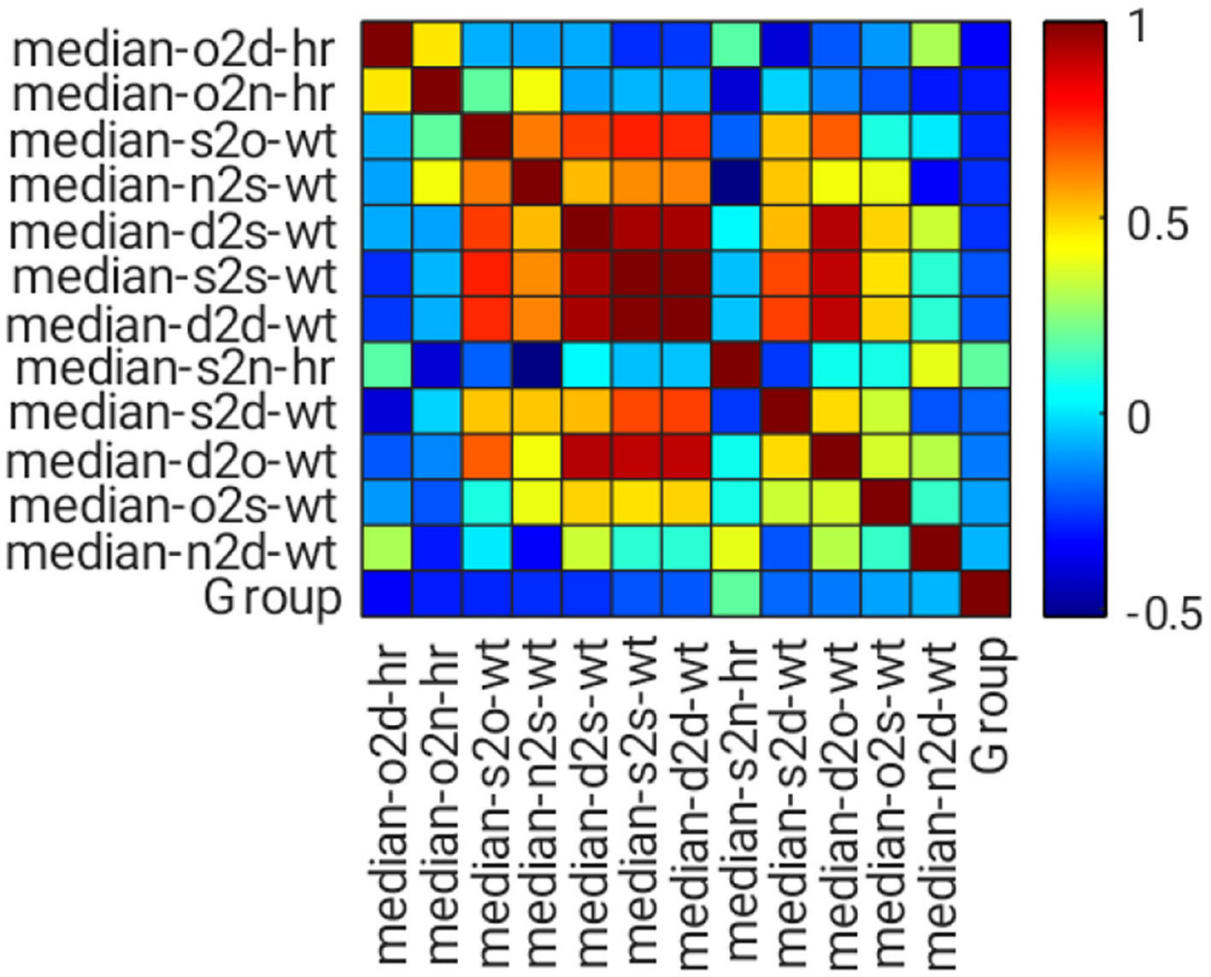
Heatmap of the correlation matrix for feature selection.

Figure 7 shows the correlation matrix among features and classification groups, as illustrated in heatmap format. Dark red, dark blue, and lighter colors indicate strongly positive, strongly negative, and weak or no correlation among the observed variables, for example, feature or group. In this figure, median-s2s-wt, median-d2d-wt, and median-d2o-wt features have a strong correlation with median-d2s-wt. Hence, only median-d2s-wt was selected because it has the highest correlation strength with the classification group.

**Figure 8:**
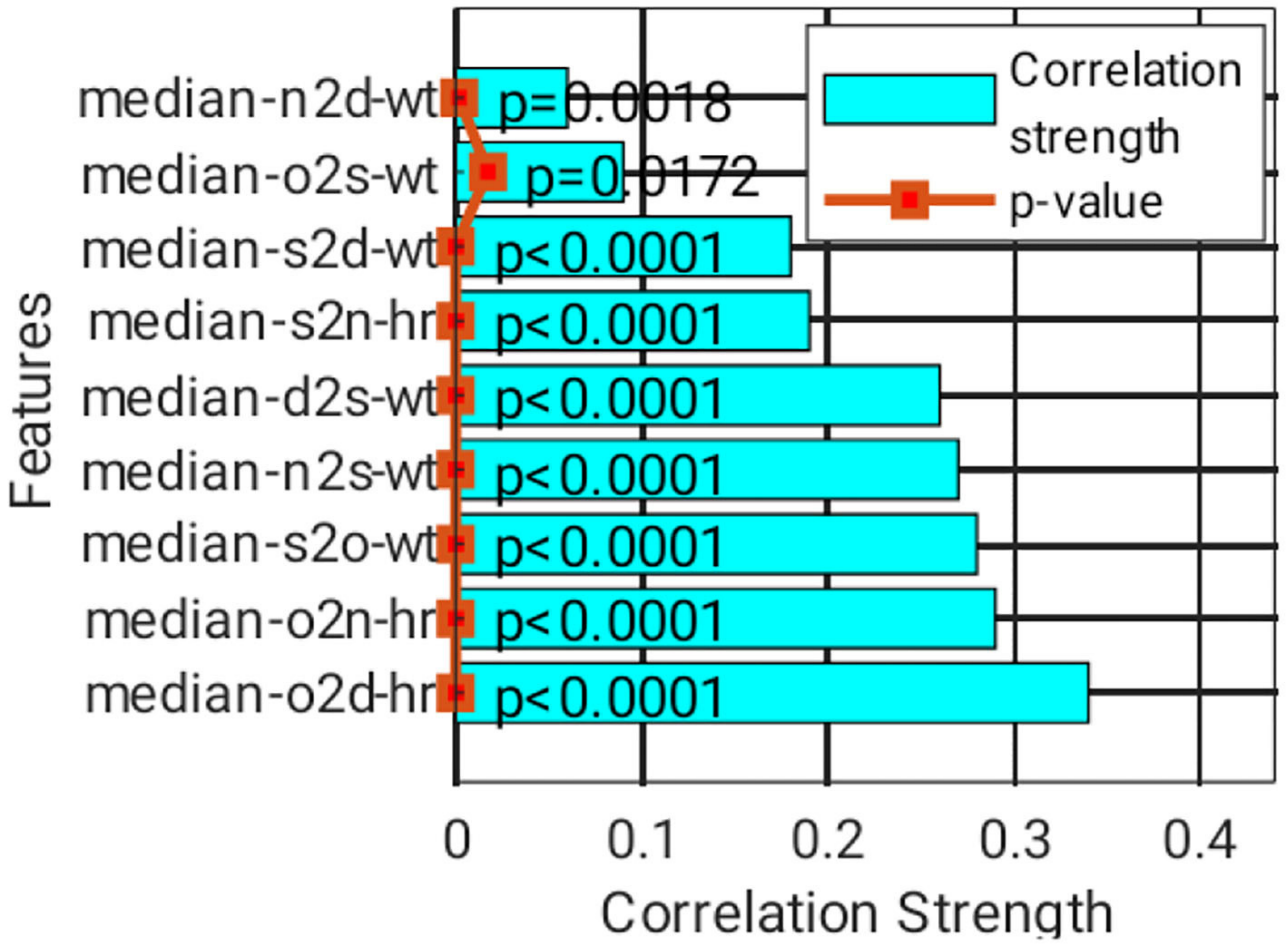
Correlation strength and *P* value of the selected features.

A total of nine features were short-listed (Figure 8), with the highest correlation strength found in the classification group, which was sorted from the bottom bar. The top three features include o2d_hr, o2n_hr, and s2o_wt. These features have a low correlation. However, their correlation with the classification group is generally weak, where at least five of them have |*r*| > 0.2, although they are statistically significant (*P* < 0.05). Thus, ML is genuinely needed.

### Machine Learning

**Figure 9:**
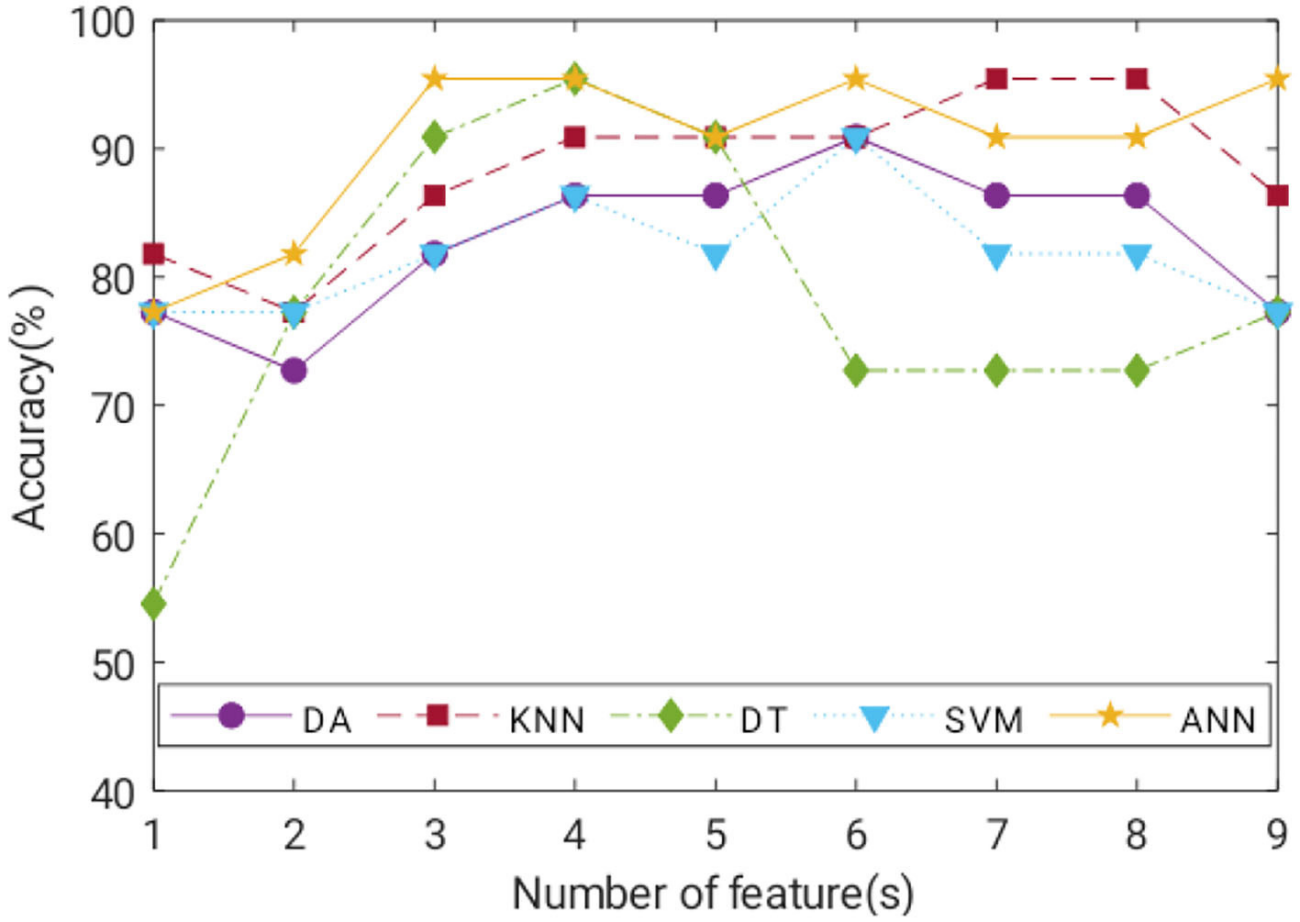
Accuracy of the best-trained model for all ML with increasing input features.

Classification of ACC for the trained model of each classifier with a number of features is illustrated in Figure 9. In general, ACC increased as more features were added, at least up to cumulative fourth features. KNN, DT, and ANN can classify negative and positive classes of COVID-19 with the highest ACC of 95.45%. SN of ANN and DT was 100%, whereas KNN achieved 100% SP. A detailed comparison of the best model performance for each ML is listed in Table 5.

**Table 5:** Performance comparison of the best-trained model for all ML.

**ML**	**DA**	**KNN**	**DT**	**SVM**	**ANN**
No. of features	6	7	4	6	6
Setting	Type = Linear	k = 7	Split = 1	Kernel = Linear	HN1 = 9 HN2 = 13
Val MSE	0.4167	0.3077	0.4615	0.3333	0.1538
Val ACC	58.33	69.23	53.85	66.67	84.62
Test MSE	0.0909	0.0455	0.0455	0.0909	0.0455
Test SP	90.91	100.00	90.91	90.91	90.91
Test SN	90.91	90.91	100.00	90.91	100.00
Test ACC	90.91	95.45	95.45	90.91	95.45
AUC	0.9174	0.9752	0.9463	0.8926	0.9587

*k, number of neighbors, Val, validation, HN, hidden nodes*.

**Figure 10:**
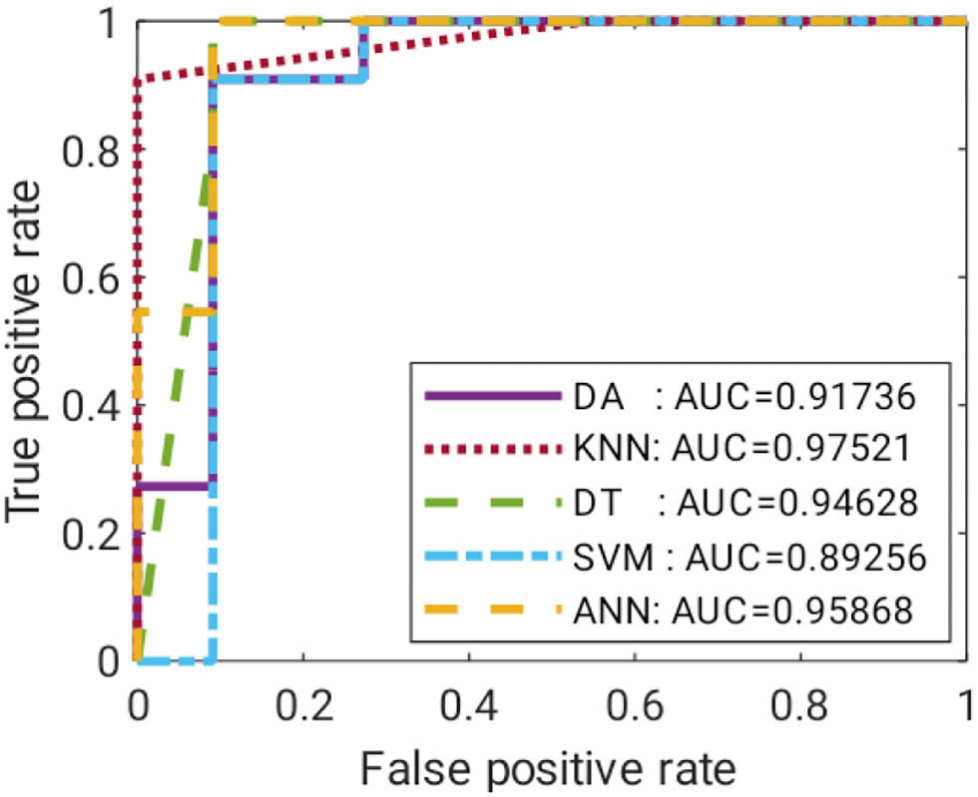
ROC curves for the best-trained model of all ML.

Satisfactory COVID-19 prediction performance was achieved using DA and SVM, where both classifiers produced 90.91% for ACC, SN, and SP using six input features. DT performed well on the test data but not on the validation data. It has the lowest validation ACC and the highest validation MSE compared with other ML models. ANN performance was excellent, where it used six features, and it had the lowest MSE, highest validation ACC, and comparable AUC to the KNN model (Figure 10). According to Figure 9, ANN performed relatively the best in six different combination features. Thus, ANN is selected as the best predictor model for COVID-19 classification.

The prediction of COVID-19 infection through PPG morphology is limited by solely using the pulse oximeter as the data recording device. Using the proposed algorithms could further develop a mobile application connected to the pulse oximeters to function as a primary diagnostic tool where the test data can be stored in a cloud for further analysis.

## Conclusion

This work pioneered the study of the relationship between COVID-19 and PPG features. ML algorithms of DA, KNN, DT, SVM, and ANN were applied for COVID-19 prediction, in which the ANN model performed remarkably to achieve 95.45% ACC, 100% SN, and 90.91% SP, using six significant features. A COVID-19 prediction method was developed using multiple PPG features extracted from a low-cost pulse oximeter.

## Data Availability Statement

The original contributions presented in the study are included in the article/supplementary files, further inquiries can be directed to the corresponding author/s.

## Ethics Statement

The studies involving human participants were reviewed and approved by Jawatankuasa Etika Penyelidikan, Universiti Kebangsaan Malaysia. The patients/participants provided their written informed consent to participate in this study.

## Author Contributions

All authors listed have made a substantial, direct, and intellectual contribution to the work and approved it for publication.

## Funding

This work was supported in part by the Institute Islam Hadhari under grant of Kursi Syeikh Abdullah Fahim RH-2020-007.

## Conflict of Interest

The authors declare that the research was conducted in the absence of any commercial or financial relationships that could be construed as a potential conflict of interest.

## Publisher's Note

All claims expressed in this article are solely those of the authors and do not necessarily represent those of their affiliated organizations, or those of the publisher, the editors and the reviewers. Any product that may be evaluated in this article, or claim that may be made by its manufacturer, is not guaranteed or endorsed by the publisher.
